# Cell Mediated Immunity in Human Pathology: The Importance of Choosing the Right Weapon

**DOI:** 10.1155/S1064744996000269

**Published:** 1996

**Authors:** Mario Clerici, Chiara Vismara, Claudia Clerici, Maria Luisa Villa

**Affiliations:** 1 Cattedra di Immunologia Polo L.I.T.A., H.L. Sacco Università degli Studi di Milano Via Venezian Milano 20133 Italy; 2 Istituto Nazionale Tumori Università degli Studi di Milano Italy; 3 Scuola di Specializzazione in Ematologia I Università degli Studi di Milano Italy


Infectious Diseases in Obstetrics and Gynecology 4:117–121 (1996)
© 1996 Wiley-Liss, Inc.

### Cell Mediated Immunity in Human Pathology: The Importance of Choosing the Right Weapon

Mario Clerici,[1] Chiara Vismara,[2] Claudia Clerici,[3] and Maria Luisa Villa[2]

*[1]Cattedra di Immunologia, Polo L.I.T.A., H.L. Sacco, and [2]Istituto Nazionale Tumori; [3]Scuola di Specializzazione in Ematologia I, Università degli Studi di Milano*

KEY WORDS

immunology; HIV infection; tumor immunology; pregnancy

The immune response tends to be regulated in a dichotomous way. A consequence of this is the observation that distinct immune effector mechanisms can be activated in response to different pathogens; these mechanisms are mainly secondary to the activation of B lymphocytes and of humoral immunity, or to the activation of cell-mediated immunity. Activation of B lymphocytes, and production of antibodies has been shown to be more useful in defense against cell-free agents and toxins (e.g., pneumococcus; tetanus toxins). In contrast, triggering of cell-mediated immunity appears to be effective against infections by cell-associated pathogens and parasites (e.g., leishmania; mycobacteria; toxoplasma; treponema; HIV).[1,2]

The mechanisms that regulate the induction of humoral and cell mediated immunity have been clarified by the identification in mice and man of two functionally distinct T helper lymphocyte compartments defined as T helper 1 (TH1) and T helper 2 (TH2).[3-5] TH1 lymphocytes secrete interferon gamma (IFNγ) and interleukin (IL)-2 and mainly promote cell mediated immunity; TH2 lymphocytes secrete IL-4, and IL-5 and mainly stimulate the humoral immunity and the generation of antibodies.[3-5] Because cytokines are produced by cell types other than T lymphocytes a functional definition was introduced; thus, type 1 cytokines are defined as those mainly stimulating cell mediated immunity whereas type 2 cytokines mainly stimulate humoral immunity. IL-2, IL-12, IFNγ, and probably IL-15 are type 1 cytokines; IL-4, IL-5, IL-6, IL-10, and IL-13 are type 2 cytokines.[6,7] We will use this functional definition throughout the review.

Analyses of different pathologic and physiologic conditions have revealed that selecting and activating the right weapon results in a better chance to control and favourably influence these conditions. In this review we will briefly summarize results obtained when we examined humoral immunity (HI) and cell mediated immunity (CMI) in conditions as different as human immunodeficiency virus (HIV) infection, neoplastic diseases, and physiologic and pathologic human pregnancy.

### IMMUNE RESPONSE IN HIV INFECTION

Defective antigen- and mitogen-stimulated IL-2 production has been described in HIV infection since 1985[8,9]. Analyses of IL-2 production showed that subtle and complex defects in T helper (TH) function are present in approximately 2/3 of HIV-seropositive asymptomatic individuals independently of a decline in CD4 counts.[10] These TH defects are predictive for: 1) rate of decline in the

Address reprint requests to Mario Clerici, MD, Cattedra di Immunologia, Università degli Studi di Milano, Via Venezian, 1, 20133 Milano, ITALY
Supported by grants from Istituto Superiore di Sanita, "VII Progetto AIDS 1993 &VIII Progetto AIDS 1994."

Received September 15, 1996
Accepted October 1, 1996

Article number of CD4$^+$ T lymphocytes;[11] 2) time to diagnosis of AIDS;[12] and 3) time to death.[12] Therefore, IL-2 production defects are predictive for three clinically relevant endpoints in HIV infection. Because in this infection IL-2 production is impaired (and IL-2 mainly stimulates CMI) and HI is abnormally activated[13] and because different cytokines stimulated CMI or HI, a number of laboratories examined type 1 and type 2 cytokine production by PBMC of HIV-seropositive individuals. The results showed that, beside IL-2, IL-12 is also severely defective in HIV infection, whereas the production of the type 2 cytokines IL-4, IL-5, IL-6, and IL-10 is enhanced.[6,7] Because different cytokines are responsible for diverse biologic effects, the concentration of IgE (IL-4-driven) and eosinophils (IL-5-driven) was analized in HIV-seropositive individuals. As expected, hyperIgE[14] and hypereosinophilia[15] were detected in those patients in whom defective (IL-2-, IL-12-, and IFNγ-driven) delayed type hypersensitivity reactions (DTH) to common recall antigens is present.[16] Additionally, hyper IgE, hypereosinophilia, and impaired DTH were recognized as predictors of poor prognosis in HIV-seropositive individuals.[14–16] These data led to the formulation of the hypothesis that a type 1-to-type 2 cytokine shift is present in HIV infection.[6,7] Corollary to the hypothesis is the prediction that strong type 1 cytokine production and well preserved CMI would correlate with lack of disease progression.

Because HIV infection results in different clinical outcomes,[17] markers of progression and protection were analyzed in HIV-seropositive individuals progressing toward AIDS and in those patients, defined as long term non-progressors (LTNP), who do not show signs of disease and maintain stable CD4 counts, despite a long-lasting HIV infection. Type 1 and type 2 cytokine production was examined in pediatric and adult cohorts to analyze similarities between vertically-transmitted and adult-acquired HIV infection. In both cohorts data obtained in LTNP were compared to those of patients with progressive HIV infection. Both in adult-acquired and vertically-transmitted infection type 1 cytokine production was significantly augmented and type 2 cytokine production was significantly reduced in HIV-seropositive LTNP individuals compared to HIV-seropositive individuals with progressive HIV infection.[18,19] Therefore, cytokine production profiles by in vitro PHA-stimulated PBMC can distinguish between HIV-seropositive patients with different patterns of disease progression in that a type 1 predominance is evident in LTNP patients whereas a type 2 predominance is characteristic of patients with progressive HIV infection. To summarize, strong CMI appears to be at least partially protective against the progression of HIV infection.

### IMMUNE RESPONSE IN NEOPLASTIC DISEASES

Type 1 cytokines have been shown to have anti-tumor and anti-metastatic effects in murine tumors. Thus: 1) IL-2, IL-12 and IFNγ activate cytotoxic T lymphocyte- and natural killers cell (NK)-mediated cytolytic functions that provide effective anti-tumor defense mechanisms;[20-22] 2) IL-2 induces the transformation of NK into lymphokine activated killers, associated with improved capacity to destroy tumor cells;[23] 3) IL-12 has direct anti-tumor activity in murine tumors.[20-22] In contrast, type 2 cytokines, and IL-10 in particular, were shown to be associated with enhanced tumor growth.[24-29] Thus, IL-10 production was reported to be elevated in certain tumors including bronchogenic carcinoma, renal cell cancer, basal and squamous cell carcinomas, lymphomas, gliomas, and melanomas.[24-29] We analyzed antigen-and mitogen-stimulated IL-2 production in newly diagnosed, untreated patients with Hodgkin's lymphoma. Additionally, we analyzed IFNγ, IL-2, IL-4, IL-10 production in women with cervical intraepithelial neoplasia of the portio.

### Hodgkin's Lymphoma

Similarly to the situation observed in HIV infection, mitogen-stimulated IL-2 production is known to be defective in patients with Hodgkin's lymphoma. IL-2 defects are accompanied by a variety of alterations in CMI including deficient DTH to common recall antigens, delayed allograft rejection, and alterations in graft versus host reactivity in vivo.[30–33] We analyzed in details IL-2 defects in newly diagnosed, untreated Hodgkin's disease patients using the same methods employed in HIV-seropositive individuals.[10] Thus, we assessed in vitro TH function by measuring IL-2 production in response to recall antigens, HLA alloantigens, and phytohemagglutinin because these different antigens are known to activate the immune response via diverse

TH-antigen presenting cell pathways.[34] Similarly to what was observed in HIV-seropositive patients, we verified that defective TH function is detected in the majority of Hodgkin's lymphoma patients.[35] Additionally we observed that the more impaired profiles of TH dysfunction were associated with a more severe clinical presentation and with less favorable hematological parameters.[35]

### Cervical Intraepithelial Neoplasia (CIN) of the Portio

Genital infection with human papillomavirus (HPV) is correlated with high risk of malignant transformation, and HPV-associated cervical intraepithelial neoplasia (CIN) of the portio is likely to be invasive.[36–39] We analyzed immune profiles in women with CIN associated with HPV infection limited to the portio or involving other sites of the lower genital tract. We observed that antigen-stimulated IL-2 production was reduced in the patients with HPV infection extended beyond the portio compared to the women with HPV infection limited to the portio.[40] Additionally, we observed that mitogen-stimulated IL-4 and IL-10 production was elevated in the former compared to the latter group.[40] Thus, a different pattern of cytokine production was observed in women with HPV infection confined to the portio (high antigen-stimulated IL-2 production; low mitogen-stimulated IL-4 and IL-10 production) compared to patients with HPV infection extending beyond the portio (low antigen-stimulated IL-2 production; high mitogen-stimulated IL-4 and IL-10 production). Briefly, we verified that a more extended and aggressive HPV infection is associated with defective type 1 cytokine production and augmented type 2 cytokine production. Thus, production of cytokines that mainly enhance potentially protective cell-mediated immunity is impaired in women in whom extended HPV infection is observed.[40]

### IMMUNE RESPONSE IN HUMAN PHYSIOLOGIC AND PATHOLOGIC PREGNANCY

Finally, because: 1) the fetus expresses human leukocyte antigens (HLA) of both maternal and paternal origin, and is thus partly allogeneic to the mother;[41] 2) despite being biological allografts, fetuses are not normally rejected by the maternal immune system; and 3) allograft rejection is classically a type 1 cytokine-dependent CMI-mediated phenomenon,[42] we analyzed type 1 and type 2 cytokines in human pregnancies. Support to the hypothesis that a type 1-to type 2 shift is observed in pregnancy stems from the observations that: 1) pregnancy in mice is a TH2-related phenomenon as a shift from predominant TH1-driven CMI to predominant TH2-driven HI is present;[43] and 2) pregnant women exhibit clinical remission of CMI-mediated autoimmune diseases such as rheumatoid arthritis as well as exacerbation of autoantibody-mediated autoimmune pathologies such as systemic lupus erythematosus, myasthenia gravis, and Graves' disease.[44–47] Support for the hypothesis that this shift could be associated with successful pregnancy derives from the following observations: 1) high concentrations of type 1 cytokines are associated with spontaneous abortions and fetal reabsorption in mice;[43] and 2) recurrent spontaneous abortion in humans is correlated with TH1 type immunity to the trophoblast.[48,49]

Again, we analyzed antigen-and mitogen-stimulated cytokine production in pregnant women undergoing physiologic pregnancy at the time the blood was drawn. Spontaneous abortions were observed in a percentage of women during follow-up, whereas a number of women delivered babies that were small for gestational age (SGA). To summarize the data, we observed that: 1) an increase in the production of type 2 cytokines by in vitro mitogen-stimulated PBMC was accompanied by a parallel decrease in the production of type 1 cytokines in successful human pregnancy, with a type 1-to-type 2 shift observed in the third trimester;[50] and 2) the absence of a type 2 bias was associated with, and possibly a predictor of, pathologic events such as spontaneous abortions and the birth of SGA babies.[50] Thus, cytokine modulation may be associated with successful human pregnancy.

### CONCLUSIONS

In this brief review we have summarized data obtained in a number of human pathologic and physiologic conditions. The results underline the importance of fighting the battle with the right weapon and suggest that choosing the wrong defense mechanism could have deleterious effects. Pharmacologic immune modulation has still not been finely tuned since most drugs induce immunosuppression rather than redirection of the immune response. A primary challenge for the future will be to learn how to teach the immune system to react to antigenic stimulation, picking the most advantageous way for the host.

### REFERENCES

1. Clerici M: Immune responses to HIV. AIDS 7: s135-s141, 1993.
2. Kaufmann SHE: Immunity to intracellular bacteria. Annu Rev Immunol 11:129–163, 1993.
3. Mosmann TR, Coffman RI: Two types of mouse T helper cell clone: Implication for immune regulation. Immunol Today 8: 223–226, 1987.
4. Mosmann TR, Coffman RI: TH1 and TH2 cells: Different pattern of lymphokine secretion lead to different functional properties. Annu Rev Immunol 7:145–168, 1989.
5. Romagnani S: Human TH1 and TH2 subsets: Doubt no more. Immunol Today 12:256–257, 1991.
6. Clerici M, Shearer GM: Is HIV associated with a TH1/TH2 switch? Immunol Today 14:107–111, 1993.
7. Clerici M, Shearer GM: The TH1/TH2 model of HIV infection: New insights. Immunol Today 15:575–581, 1994.
8. Lane HC, Depper JM, Greene WC et al.: Qualitative analysis of immune function in patients with the acquired immunodeficiency syndrome: Evidence for a selective defect in soluble antigens recognition. N Eng J Med 313:79-84, 1985.
9. Smolen JS, Bettleheim P, Koller U et al.: Deficiency of the autologous mixed lymphocyte reaction in patients with classic hemophilia treated with commercial Factor VIII concentrate: Correlation with T cell subset distribution, antibodies to lymphadenopathy-associated or human T lymphotropic virus, and analysis of the cellular basis of the deficiency. J Clin Invest 75:1828–1834, 1985.
10. Clerici M, Stocks NI, Zajac RA et al.: Detection of three distinct patterns of T helper cell dysfunction in asymptomatic, human immunodeficiency virus-seropositive patients. Independence of CD4$^+$ cell numbers and clinical staging. J Clin Invest 84:1892–1899. .1989
11. Lucey DR, Melcher GP, Hendrix CW, and the USAF HIV Study Group: The U.S. Air Force HIV study 1985–1990: Immunological analyses, seroconversion and the potential utility of a T-helper functional assay to predict change in CD4$^+$ T-cell counts during early stage HIV infection J Infect Diseases 164:631–637, 1991.
12. Dolan MJ, Clerici M, Blatt SP et al.: In vitro T cell function, delayed type hypersensitivity skin testing, and CD4 T cell subset phenotyping independently predict survival time in patients infected with human immunodeficiency virus. J Infect Dis 172:79–87, 1995.
13. Levy JA. Pathogenesis of human immunodeficiency virus infection: Microbiol Rev 57:183–233, 1993.
14. Israel-Biet D, Labrousse F, Tourani J-M, Sors H, Andrieu JM, Even P: Elevation of IgE in HIV-infected subjects: A marker of poor prognosis. J Allergy Clin Immunol 89:68–75, 1993.
15. Smith KJ, Skelton HG, Drabick JJ, McCarthy WF, Ledsky R, Wagner KF: Hypereosinophilia secondary to immunodysregulation in patients with HIV-1 disease. Arch Dermatol 130:119–121, 1994.
16. Blatt SP, Hendrix CW, Butzin CA et al.: Delayed type hypersensitivity skin testing predicts progression to AIDS in HIV-infected patients. Ann Int Med 119:177–184, 1993.
17. Levy JA: HIV pathogenesis and long-term survival. AIDS 7:1401–1410, 1993.
18. Clerici M, Balotta C,Meroni L et al.: Type 1 cytokine production and low prevalence of viral isolation correlate with long term non progression in HIV infection. AIDS Res Hum Retrovir 12:1053–1061, 1996.
19. Vigano' A, Balotta C,Trabattoni D et al.: Immunology and virology of long term resistance to HIV in vertical HIV infection: Characterization of long term resistant hosts. Submitted
20. Brunda MJ, Luistro L, Warrier RB et al.: Antitumor and antimetastatic activity of interleukin-12 against murine tumors. J Exp Med 178:1223–1230, 1993.
21. Tahara H, Zitvogel L, Storkus WJ et al.: Effective eradication of established murine tumors with IL-12 gene therapy using a polycistronic retroviral vector. J Immunol 154:6466–6474, 1995.
22. Gateley MK, Warrier RR, Honasoge S et al.: Administration of IL-12 to normal mice enhances cytolytic lymphocyte activity and induces production of IFN gamma in vivo. Int Immunol 6:157–167, 1994.
23. Trinchieri G: Biology of natural killer cells. Adv Immunol 47:187–376, 1989.
24. Bost KL, Bieligk SC, Jaffe BM: Lymphokine mRNA expression by tranplantable murine B lymphocytic malignancies. Tumor derived IL-10 as a possible mechanism for modulating the anti-tumor response. J Immunol 154:718–729, 1995.
25. Kim J, Modlin RL, Moy RL, et al.: IL-10 production in cutaneous basal and squamous cell carcinomas. A mechanism for evading the local T cell immune response. J Immunol 155:2240–2247, 1995.
26. Smith DR, Kunkel SL, Burdick MD et al.: Production of IL-10 by human bronchogenic carcinoma. Am J Pathol 145:18–25, 1994.
27. Maeurer MJ, Martin DM, Castelli C et al.: Host immune response in renal cell cancer: IL-4 and IL-10 mRNA are frequently detected in freshly collected tumor-infiltrating lymphocytes. Cancer Immunol Immunother 41:111–121, 1995.
28. Huettner C, Paulmus W, Roggendorf W: Messenger RNA expression of the immunosuppressive cytokine IL-10 in human gliomas. Am J Pathol 146:317–322, 1995.
29. Chen Q, Daniel V, Maher DW, Hersey P: Production of IL-10 by melanoma cells: Examination of its role in immunosuppression mediated by melanoma. Int J Cancer 56:755–760, 1994.
30. Young RC, Corder MP, Haynes HA, DeVita VT: Delayed hypersensitivity in Hodgkin's disease. Am J Med 52:63–71, 1972.

31. Moghe MV, Advani SH, Gangal SG: Demonstration of inhibitory factors affecting cell-mediated immunity in patients with Hodgkin's disease. Eur J Cancer 16:937–941, 1980.
32. Soillou JP, Douillard JY, Vie H, et al.: Defect in lectin-induced IL-2 production by peripheral blood lymphocytes of patients with Hodgkin's disease. Eur J Cancer Clin Oncol 21:935–942, 1985.
33. Zamkoff KW, Reeves WG, Paolozzi FP et al.: Impaired interleukin regulation of the PHA response in Hodgkin's disease. Clin Immunol Immunopathol 35:111–124., 1985
34. Via CS, Tsokos GC,Stocks NI, et al.: Human in vitro allogeneic responses: Demonstration of three pathways of T helper cell activation. J Immunol 44:2524–2229, 1990.
35. Clerici M, Ferrario E, Trabattoni D et al.: Multiple defects of T helper cell function in newly diagnosed patients with Hogkin's disease. Eur J Cancer 30:1464–1470, 1994.
36. Ferenczy A: Epidemiology and clinical pathophysiology of condilomata acuminata. Am J Obster Gynecol 172:1331–1339, 1995.
37. Wright TC, Richart R: Role of HPV in the pathogenesis of genital tract warts and cancer. Gynecol Oncol 37:151-164, 1990.
38. Gissman L: Human papillomaviruses and genital cancer. Seminar Cancer Biol 3:253–261, 1992.
39. Lorincz AT, Reid R, Jenson AB, Greenberg MD, Lancaster W, Kurman RJ: Human papillomavirus infection of the cervix: Relative risk association of 15 common anogenital types. Obstet Gynecol 79:328–337, 1992.
40. Clerici M, Merola M, Ferrario E et al.: Cytokine production patterns in cervical intraepithelial neoplasia: Association with HPV infection limited to the portio or involving other sites of the lower genital tract. JNCI (in press).
41. Abbas AK, Lichtman AH, Pober JS: Cellular and Molecular Immunology 2nd. edition. Philadelphia: W.B. Saunders Co. 1994, p 354.
42. Lafferty KJ, Prawse SJ, Simeonovic RJ, Warren HS: Immunobiology of tranplantation. Annu Rev Immunol 1:143–173, 1983.
43. Wegmann TG, Lin H, Guilbert L, Mosman TL: Bidirectional cytokine interactions in the maternal-fetal relationship: Is successful pregnancy a TH2 phenomenon? Immunol Today 14:353–358, 1993.
44. Da Silva J A, Spector TD: Rheumatoid arthritis in pregnancy. Clin Rheumatol 11:189–202, 1992.
45. Nossent JC: Systemic Lupus Erythematosus: IV. Analysis of the interrelationship with pregnancy. J Reumatol 17:771–778, 1990.
46. Fennel DF, Ringell SB: Myastenia gravis and pregnancy. Obstet Gynec Survey 42:414–421, 1987.
47. Kendall-Taylor P: Pregnancy and the thyroid. Fet Mat Med Rev 5:89–103, 1993.
48. Hill JA, Polgar K, Anderson DJ: T-helper 1-type immunity to trophoblast in women with recurrent spontaneous abortion. JAMA 273:1933–1937, 1995.
49. Dudley D: Recurrent pregnancy loss and cytokines. JAMA 273:1958, 1995.
50. Marzi M, Vigano' A, Trabattoni D, Villa ML, Clerici E, Clerici M: Characterization of type 1 and type 2 cytokine production profile in physiologic and pathologic human pregnancy. Clin Exp Immunol 106:127–133, 1996.
